# APOE2 reduces risk of Alzheimer's disease by protection of lysosomes from lipid overloading

**DOI:** 10.1016/j.apsb.2025.03.001

**Published:** 2025-03-06

**Authors:** Jing Feng, Xiwen Ma, Jianping Ye

**Affiliations:** a Institute of Trauma and Metabolism, Zhengzhou Central Hospital Affiliated to Zhengzhou University, Zhengzhou 450007, China; b Tianjian Laboratory of Advanced Biomedical Sciences, Academy of Medical Sciences, Zhengzhou University, Zhengzhou 450001, China

**Keywords:** APOE2, APOE3, APOE4, Lipofuscin, LDLR, Alzheimer's disease

The Apolipoprotein E (*APOE*) genotype is closely associated with risk of Alzheimer's disease (AD), a progressive neurodegenerative disorder that impairs cognitive functions of the brain. APOE2 reduces while APOE4 increases the risk of AD. Extensive research has been conducted to understand the differences among APOE isoforms in the pathogenesis of AD^1^. However, the exact molecular mechanism underlying APOE isoform actions remains unclear. A recent study published in *Cell* brought new insights into the mechanism^2^, revealing that APOE2 reduced interactions between lipidated APOE (lipAPOE) and low-density lipoprotein receptor (LDLR), thereby conferring protection to lysosomes in astrocytes and neurons.

APOE is the most abundant apolipoprotein in the brain, facilitating lipid transport between cells. It has three major isoforms: APOE2, APOE3, and APOE4. APOE is primarily expressed by astrocytes, neurons, and reactive microglia, and its receptor, LDLR, is expressed in various cell types, especially in excitatory neurons in the human brains^3^. APOE is implicated in regulating several key pathological events in AD, including amyloid-*β* (A*β*) aggregation, tau pathology, and lysosomal trafficking, all of which collectively influence the risk of AD^4^. In mechanism, APOE may induce these effects by regulating lipid metabolism. APOE4 contributes to AD by disrupting cholesterol metabolism, including cholesterol transport, esterification, and the expression of cholesterol metabolism genes^1^^,^^5^. Over the past three decades, most studies have focused on elucidating the mechanisms of APOE4 activity in AD pathogenesis, while less investigation was made in the mechanism associated with APOE2. In 1982, APOE2 was found to exhibit low activity in binding to LDLR^6^. Similarly, an *APOE3* mutant *(APOE3*-R136S or Christchurch variant), was found to exhibit 60% reduction in LDLR binding activity in its homozygous form, which was shown to suppress both amyloid and non-amyloid pathology in the mouse model of AD^7^. Individuals carrying two copies of the mutant were protected from cognitive impairment despite amyloid deposition in the brain, which exhibited a low level of tau pathology with a remarkable 30-year delay in dementia onset^8^. The shared characteristics between APOE2 and the APOE3 Christchurch variant have led to a hypothesis that reduced binding of lipAPOE to LDLR could be a protective mechanism against AD^2^.

To test the hypothesis, the authors of new study utilized homogeneous time-resolved fluorescence (HTRF) and surface plasmon resonance (SPR) assays to demonstrate the binding activity of lipAPOE2 to LDLR for endocytosis^2^. Using pHrodo-labeled APOE-high-density lipoprotein (HDL), they observed that the efficiency of lipoprotein uptake was lowest for APOE2. LipAPOE2 significantly reduced the interaction of lipAPOE3/E4 with LDLR, resulting in decreased lipid uptake by cells, highlighting lipAPOE2 in the regulation of lipoprotein binding to LDLR during lipid uptake (Fig. 1). Additionally, LDLR-mediated lipoprotein uptake was influenced by cholesteryl ester (CE). Specifically, CE species containing polyunsaturated fatty acids (PUFAs), such as CE (20:4), exhibited an increase in lipoprotein uptake. Super-resolution imaging revealed co-localization of clustered LDLR with APOE within LAMP^+^ (lysosomal marker) compartments^2^, indicating that the lipoproteins were directed to the endo-lysosomal system after entering cells. In neurons derived from the pluripotent stem cells, CE (20:4)-containing lipAPOE had higher levels of lipid deposition within lysosomes. Lipofuscin, a hallmark of age-related lysosomal dysfunction, arises from lipid peroxidation and is associated with neuronal aging for a high risk of AD.

**Figure 1:**
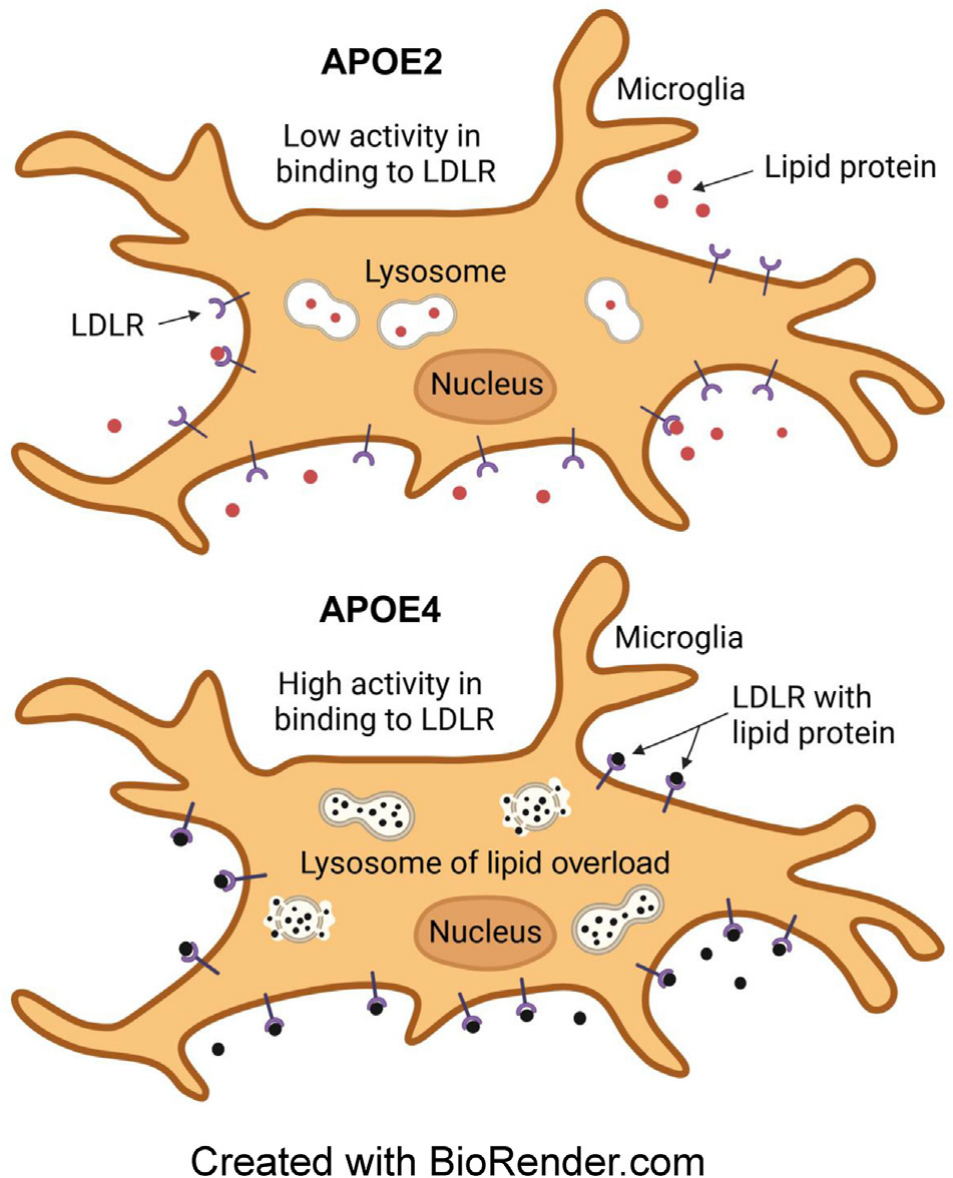
The different binding capacities of APOE2 and APOE4 to LDLR affect lysosomal lipid load (By biorender).

Isoform-specific induction of lipofuscin was observed in cultured cells in the following order: APOE4>APOE3>APOE2. Lipofuscin accumulation required LDLR-mediated lipoprotein endocytosis. Contrary to APOE2, APOE4 exhibited a higher activity to induce lipofuscin accumulation in human astrocytes and neurons. Lipofuscin accumulation was observed in *APOE4* knock-in (KI)/P301S *MAPT* mice^5^. This activity of APOE4 was further augmented by PUFA, as PUFA-CE-lipAPOE4 robustly induced lipofuscin formation *in vivo*, facilitating tau fibril accumulation. These findings suggest that LDLR-mediated endocytosis of lipoprotein particles was delivered to lysosomes, enhanced by APOE3/E4 isoforms and PUFAs, leading to lysosomal degeneration with lipofuscin accumulation. Treatment with liver X receptor agonists, which reduce PUFA-CE levels, significantly inhibited lipofuscin accumulation and lysosomal degeneration in the mouse model. APOE2 and the Christchurch variant prevented lysosomal degeneration by reducing lipoprotein uptake and lysosomal lipid loading.

The new study extends the mechanism of APOE impact on lysosomes from previous studies. In 2017, APOE4 was found to associate with lipofuscin accumulation in the AD-associated brain regions in a tauopathy mouse model^9^. In the study, APOE4 with PUFA-CE induced lipofuscin accumulation both *in vitro* and *in vivo*, linked to lipid peroxidation and lysosomal dysfunction in astrocytes and neurons. In 2020, Rongcan Luo et al.^10^ demonstrated that upregulation of PPAR*α*-mediated autophagy of lysosomes could reduce amyloid pathology. The study suggested that lipofuscin accumulation in lysosomes within astrocytes and neurons exacerbates the progression of AD, while reducing lysosomal lipofuscin accumulation may delay AD onset. Decreasing interaction of LDLR and APOE2 may free the receptor to promote clearance of other ligands like A*β*, thereby reducing amyloid pathology. These studies indicate that the interaction between lipAPOE and LDLR plays a crucial role in regulating lysosomal activity in AD pathology.

In summary, the new study reveals that APOE/LDLR interaction regulates lysosome lipid metabolism. The decreased interaction between APOE2 and LDLR reduced AD risk by preventing lipofuscin accumulation in lysosomes^2^. The *APOE3* mutants (*APOE3* p.V236E, or Jacksonville variant) act in the same way to reduce amyloid burden through lower binding activities to LDLR^11^. The finding of lipid cargoes impact in the lysosomes of astrocytes and neurons extends the mechanism for A*β* aggregation in AD^12^. In addition, the APOE/LDLR interaction is enhanced by PUFA in the APOE particles. Blocking the lipAPOE4/LDLR interaction in astrocytes and neurons could potentially mitigate the risk of AD. Transforming APOE4 into variants like APOE2 presents a novel therapeutic strategy for individuals with the *APOE4* genotype. However, the findings did not confirm the lipAPOE/LDLR interaction-induced lipofuscin accumulation in oligodendrocytes, endothelial cells, and other brain cell types. Lipofuscin might be phagocytosed by microglia or astrocytes after significant accumulation. The new study suggests that lysosomal lipofuscin accumulation is an important event in the pathogenesis of AD, and a potential target for developing new therapeutic approaches.

## Author contributions

Jing Feng drafted the manuscript. Xiwen Ma and Jianping Ye provided the idea and revised the manuscript.

## Conflicts of interest

The authors declare no conflicts of interest.

## References

[1] Blanchard J.W., Akay L.A., Davila-Velderrain J., von Maydell D., Mathys H., Davidson S.M. (2022). APOE4 impairs myelination *via* cholesterol dysregulation in oligodendrocytes. Nature.

[2] Guo J.L., Braun D., Fitzgerald G.A., Hsieh Y.T., Rougé L., Litvinchuk A. (2025). Decreased lipidated ApoE-receptor interactions confer protection against pathogenicity of ApoE and its lipid cargoes in lysosomes. Cell.

[3] Mathys H., Peng Z., Boix C.A., Victor M.B., Leary N., Babu S. (2023). Single-cell atlas reveals correlates of high cognitive function, dementia, and resilience to Alzheimer's disease pathology. Cell.

[4] Windham I.A., Cohen S. (2024). The cell biology of APOE in the brain. Trends Cel Biol.

[5] Litvinchuk A., Suh J.H., Guo J.L., Lin K., Davis S.S., Bien-Ly N. (2024). Amelioration of Tau and ApoE4-linked glial lipid accumulation and neurodegeneration with an LXR agonist. Neuron.

[6] Weisgraber K.H., Innerarity T.L., Mahley R.W. (1982). Abnormal lipoprotein receptor-binding activity of the human E apoprotein due to cysteine-arginine interchange at a single site. J Biol Chem.

[7] Quiroz Y.T., Aguillon D., Aguirre-Acevedo D.C., Vasquez D., Zuluaga Y., Baena A.Y. (2024). APOE3 christchurch heterozygosity and autosomal dominant Alzheimer's disease. N Engl J Med.

[8] Arboleda-Velasquez J.F., Lopera F., O'Hare M., Delgado-Tirado S., Marino C., Chmielewska N. (2019). Resistance to autosomal dominant Alzheimer's disease in an APOE3 Christchurch homozygote: a case report. Nat Med.

[9] Shi Y., Yamada K., Liddelow S.A., Smith S.T., Zhao L., Luo W. (2017). ApoE4 markedly exacerbates tau-mediated neurodegeneration in a mouse model of tauopathy. Nature.

[10] Luo R., Su L.Y., Li G., Yang J., Liu Q., Yang L.X. (2020). Activation of PPARA-mediated autophagy reduces Alzheimer disease-like pathology and cognitive decline in a murine model. Autophagy.

[11] Liu C.C., Murray M.E., Li X., Zhao N., Wang N., Heckman M.G. (2021). *APOE3*-Jacksonville (V236E) variant reduces self-aggregation and risk of dementia. Sci Transl Med.

[12] Martens Y.A., Zhao N., Liu C.C., Kanekiyo T., Yang A.J., Goate A.M. (2022). ApoE cascade hypothesis in the pathogenesis of Alzheimer's disease and related dementias. Neuron.

